# Real-Time Documentation of the Effect of Onabotulinumtoxin A Detrusor Injection in OAB Patients—Preliminary Results

**DOI:** 10.3390/toxins15010030

**Published:** 2022-12-30

**Authors:** Heinrich Schulte-Baukloh, Catarina Weiß, Sarah Weinberger, Mandy Hubatsch, Thorsten Schlomm, Bernhard Ralla

**Affiliations:** 1 Department of Urology, Charité Universitätsmedizin Berlin, 10117 Berlin, Germany; 2 Urologic Practice, Kurfürstendamm 139, 10711 Berlin, Germany

**Keywords:** overactive bladder, botulinum toxin, urinary incontinence, urinary bladder, injections, intramuscular

## Abstract

Introduction: Detrusor injection with onabotulinumtoxin A (OnabotA-DI) is an established therapy for overactive bladder (OAB). Little is known about the exact onset and course of the effect in the days after the injection therapy. By using a new type of app-controlled automated diary pod, for the first time, the precise onset of the effect of OnabotA-DI can be documented in real time. Materials and methods: Patients due for OnabotA-DI were asked to document voiding 3 days before and up to 3 weeks after therapy using the Diary Pod app. The detrusor injection was performed with onabotulinumtoxin A (Botox^®^), 100 units, at 20 sites of the detrusor muscle in a standardized manner. Voiding on the injection day itself was not documented. Results: A total of 17 patients (15 women, 2 men; aged 33–83 (mean 64.6; median 70) years) were included in the study. The handling of the Diary Pod app was user-friendly, and elderly patients did not encounter technical problems. The results of patients with reliably documented micturitions showed a continuous reduction in micturition frequency every day from the first day and significantly from day 5. For 24 h voiding, from 12.83 ± 5.54 in the 3 days before injection, the following mean values were found with significant (*p* < 0.05) changes after the intervention: 9.17 ± 3.19 on day 5, 8.75 ± 3.69 on day 10, 7.17 ± 2.04 on day 15, and 5.75 ± 0.5 on day 20. These changes were in similar proportions during the daytime and nighttime. Conclusions: Contrary to previous knowledge, the effect of the OnabotA-DI set in from the first postoperative days and was reflected a similar extent in day and night micturition. This study is the first to document the onset of action of OnabotA-DI in real time.

## 1. Introduction

As defined by the International Continence Society [1], the overactive bladder (OAB) syndrome, urge syndrome, or urgency-frequency syndrome can be described by the presentation of urgency, with or without urge incontinence, usually with frequency and nocturia. These symptom combinations are suggestive of urodynamically demonstrable detrusor overactivity but can be due to other forms of urethro-vesical dysfunction. These terms can be used if there is no proven infection or other obvious pathology (e.g., bladder stones, bladder tumors, bladder outlet obstruction due to benign prostatic hyperplasia, or uterovaginal descenus). OAB is purely a patient-reported condition. These OAB symptoms can substantially limit the quality of life and cause various social, work-related, psychological, and sexual problems [2,3,4]. Epidemiological studies from Europe and the US have shown that the symptoms of OAB increase significantly with age and can occur in up to 16–17% of the population [5,6,7]. According to the current European Association of Urology (EAU) guidelines [8,9], behavioral therapy approaches such as weight loss, reducing nicotine and coffee consumption, pelvic floor therapy, and bladder training are therapeutic options. However, OAB is a domain of drug approaches such as anticholinergics or β_3_-receptor agonists such as mirabegron [8,9]. If these medications are ineffective or discontinued because of side effects [10], the patient can be offered more invasive measures as second-line therapy.

In 2013, onabotulinumtoxin A detrusor injection (OnabotA-DI) was approved by the US Food and Drug Administration for the treatment of idiopathic OAB in patients whose urgency or frequency with urge incontinence did not respond adequately to anticholinergic drugs or who were intolerant to these drugs [11]. According to the EMBARK study reported by Nitti et al. [11], this therapy is carried out as part of a cystoscopy procedure and provides effective alleviation of all OAB symptoms after 12 weeks [11], to the following extent: a 47.9% reduction in urinary urge incontinence, 16.9% reduction in frequency, 31.6% reduction in urgency, and 20.2% reduction in nocturia. Clinically meaningful improvements from baseline in all I-QOL and KHQ multi-item domains indicated a positive impact on HRQOL [4]. This therapeutic approach has proven to be more effective in reducing urge incontinence than oral medication [12,13]. Numerous, generally similar data exist on the duration of the effect of onabotulinumtoxin A in the urinary bladder [14]. The effect of 100 U of onabotulinumtoxin A in a long-term study by Nitti et al. based on the time to patient re-treatment request showed a median of 7.6 months, but this varied greatly [15]: 34.2% of the patients requested re-treatment within 6 months, 37.2% after 6–12 months, and 28.5% after more than 12 months.

However, data are sparse for this approach concerning the exact onset of action in the days after the OnabotA-DI therapy, and we are not aware of any study that has explicitly dealt with this issue. The drug information and numerous studies on this topic state only that “a clinical improvement generally occurs within one to two weeks”, but when exactly can the patient expect the first relief from the burdensome voiding frequency? By using a novel app-controlled automated urine collection device (Minze-Health^®^ Diary Pod), we investigated the exact onset of the effect of OnabotA-DI in real time.

## 2. Results

A total of 17 patients were included in the study (15 women and 2 men). The mean/median age was 64.6/70 (33–83) years. Unfortunately, the dropout rate was high; four patients had to be excluded due to insufficient documentation (≤6 documented postoperative days), two due to postoperative UTIs, one due to device failure, and one due to cancer (non-urological) diagnosed during the study period. On the other hand, nine very well-documented pre- and post-interventional datasets were evaluated.

**Figure 1:**
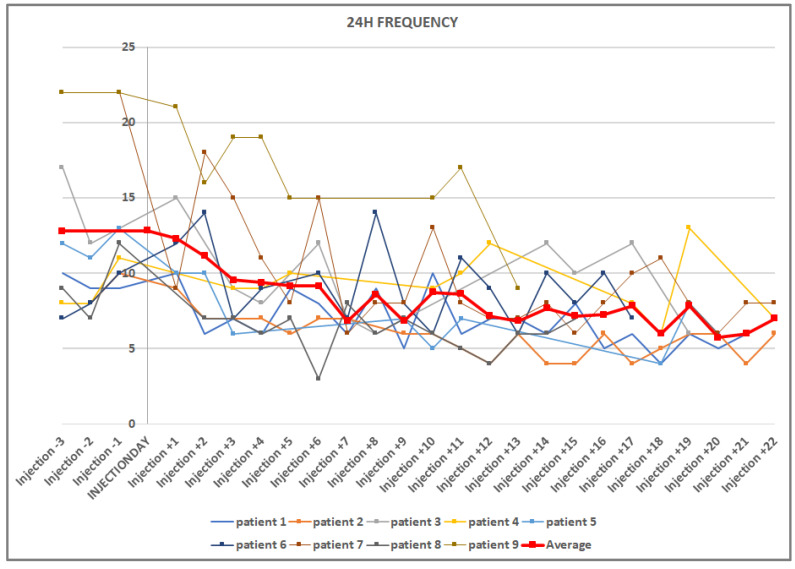
Graphic representation of the decrease in micturition frequencies in 24 h, before and after injection. The vertical line crosses on the day of injection. The mean value curve is in bold red.

The baseline mean micturition frequency in the 3 days before the injection therapy was 12.8 ± 5.54 micturitions/24 h. Figure 1 shows the decrease in micturition frequency over the 3 weeks. Table 1 shows the decrease in voiding frequency until a significant decrease was achieved, and then every 5 days thereafter to day 20. Due to the generally high variability, the mean micturition volume showed a delayed significant increase, from an average of 194.8 mL before the injection to 244.1 mL on the ninth day after the injection.

## 3. Discussion

The patients evaluated in this study were representative of those typically affected with OAB symptoms and are comparable to those in larger series (e.g., the approval EMBARK study of Nitti et al. [11]). Although our patient series was small, the patients were especially homogenous and displayed typical features. Therefore, the sample was representative of average OAB patients: the increased micturition frequency of 12.8/24 h was similar to that of the large series, as was the unequal gender distribution strongly in favor of women (89.3%), and the mean age of 64.6 years [11,16]. The frequency of post-interventional UTIs in our series at 11.8% (2 of 17) fits into the variable picture described in the literature of UTI rates of 0.4–24.5% [11,16]. In our study, these patients had to be excluded because a UTI changes the micturition frequency significantly; accordingly, this would not reflect the effect of the onabotulinumtoxin A. The considerable effort for the patients of the daily permanent documentation and Diary Pod use also led to many dropping out. Unfortunately, one patient had a diagnosis of breast cancer during the study period and did not want to continue with the study, and one device did not function properly. However, despite this high dropout rate, the results properly reflect the clinical picture of OAB, and thus, they also reflect the course of action of the onabotulinumtoxinA therapy because of the high homogeneity of the representative participants. Furthermore, this study was not a proof-of-action study, but it does prove the onset of action for onabotulinumtoxinA after application in the detrusor muscle.

Effective therapeutic results of OnabotA-DI are well-documented in patients with OAB [14]. The duration of action has also been clarified: in the long-term follow-up study reported by Nitti et al. [15], the overall median duration of the effect of onabotulinumtoxinA 100 U was 7.6 months. As mentioned, the median duration was 6 months or less in 150 of the 438 patients (34.2%), between 6 and 12 months in 163 patients (37.2%), and greater than 12 months in 125 patients (28.5%). Moreover, to our knowledge, the onset of the toxin’s effect has not been reported. This is the most important finding in our study: the frequency of voiding per 24 h decreased significantly from the fifth post-interventional day. Considering the absolute numbers, this also corresponds to a clinically substantial decrease in micturition frequency: from 12.8 to 9.2, i.e., −3.7 (−28.6%). In the pivotal study by Nitti [11], this value was −16.9% at week 12 post-treatment.

The good effect of onabotulinumtoxin A on urinary bladder symptoms is convincing. Its action is complex, especially with the most clinically used serotype botulinum toxin type A; the neuromuscular transmission is inhibited in not only efferent but also afferent nerves [17]. In the bladder, SV2 receptors, via which the neurotoxin is taken up into the nerve cells [18], and SNAP25, which is part of the SNARE protein complex and the primary target of botulinum toxin type A [19], are found in abundance and co-localize in parasympathetic, sympathetic, and sensory nerves. Almost all nerves of the cholinergic system (95%) present the SV2 receptor, as do 69% of the sympathetic and 58% of the sensory fibers [20,21]. The exact site of action in the urinary bladder is debated: whether exclusively or predominantly in the detrusor muscle and the suburothelial connective tissue or whether the urothelium could already be the site of attack of the botulinum toxin. While the SV2 receptor could be detected [22], SNAP25 was not detected in the human urothelium [20]. The inhibitory effect on the transmitter release of acetylcholine and, thus on, muscular overactivity is an important point of attack for botulinum toxin A. However, the release of several other transmitters after botulinum toxin injections are suspected in animal studies, e.g., ATP in healthy [22] and spinalized [23] animals. This could lead to a downregulation of the purinergic component at the suburothelial [24] and detrusor level [25]. However, the downregulation of the purinergic system is likely associated with reduced afferent activation and signaling since the decrease in suburothelial purinergic P2X3 receptors correlated well with the reduction in pathological sensations of urgency in patients [26]. Additionally, suburothelial capsaicin TRPV1 receptors, which play a role in the afferent mechano-sensation and pain pathways of the urinary bladder sensitization, were found to be downregulated after OnabotA-DI [24].

For the first time, our study documented precisely how the effect unfolds clinically—although no direct neurophysiological proof of the effect could be provided, of course (this would necessitate basic science investigations). However, regarding the onset of the effect of OnabotA-DI, all studies have only noted that it can be expected after around 1–2 weeks. Studies on the exact onset of action are rare. In striated muscle, Hamjian et al. [27] showed in the human M. extensor digitalis brevis that after injection of 10U onabotulinumtoxin A, neurophysiologically detectable activity decreased from 48 h, with a maximum decrease after 21 days. To our knowledge, no studies exist on the exact onset of action on the bladder or any other smooth muscle in humans. However, the data from our study are very helpful in the informational discussion because patients otherwise often expect an immediate onset of action.

## 4. Conclusions

Our study reveals for the first time the exact onset of the action of OnabotA-DI, using the example of voiding frequency because this was the most reliable documented parameter in the Diary Pod app. A trend of reduction can be seen from the 4th day, and a significant and clinically meaningful change and alleviation can be expected from the 5th day after OnabotA-DI. This is important information for the expectations of patients who choose this minimally invasive therapy.

## 5. Materials and Methods

Adult patients with OAB symptoms, as described, were included in this study. All patients had received oral anticholinergic or β_3_-receptor agonist premedication, but this was discontinued because of insufficient effect or significant side effects, or contraindications existed. OnabotA-DI was recommended. In order to make a per se good OnabotA-DI effect likely, the EAU guideline recommendation [8,9] for the diagnosis of OAB and the exclusion of other potential causes of the symptoms were carefully observed. This was performed meticulously to minimize the rate of potential treatment failures due to OnabotA-DI misindication, which would lead to misinterpretations of the onset of action. Another inclusion criterion was the presence and confident use of a smartphone (subjective assessment by the patients) to be able to load the Minze-Health app. The patients were instructed in this app and also received instructions for use in their native language. The procedure was scheduled for 2–4 weeks after this briefing.

**Figure 2:**
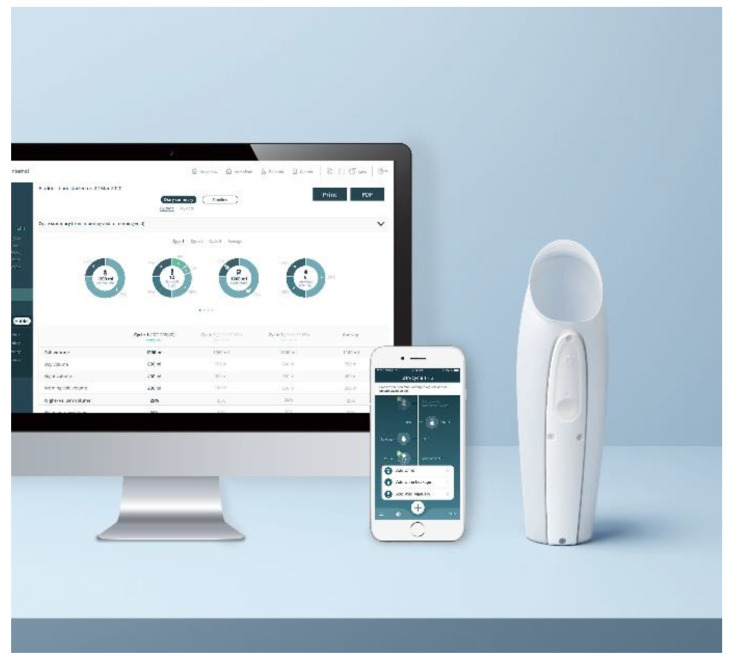
The Diary Pod, an automated bladder diary solution that includes a measuring device connected to a mobile app. The collected data are visualized in a dashboard on the Minze Clinician Portal.

**Figure 3:**
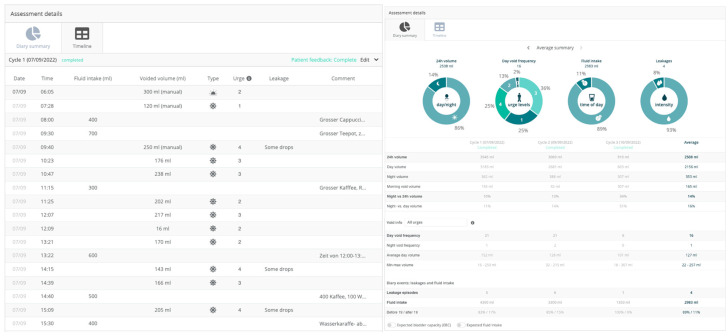
(Left side) Example of documentation of the kind and amount of drinking, micturitions, urgencies, and leakages and (right side) the summaries of these values.

The patients began using the Diary Pod app 3 days before the agreed injection date to collect the pre-therapeutic basic data. The Minze Diary Pod (Figure 2) is an automated bladder diary solution that includes a measuring device connected to a mobile app. The Diary Pod is a device for all ages and genders that measures volume and time, and the app assists the patient in keeping a bladder diary in a compliant way. The collected data are visualized in a dashboard on the Minze Clinician Portal (Figure 3 left and right graph), where the results can be analyzed, compared over time, or exported as raw data for further research. The first morning void must be marked to stop the counting of nocturnal voids (nocturia). Voiding related to defecation could not be documented and had to be ignored. In principle, patients can also document drinking quantities and episodes of urinary incontinence in the app, but this was not the primary aim of the study since we had to assume that the manual registration of incontinence events in the app documentation could be incomplete.

On the day of the injection (day 0), no micturition was documented. A urine test was performed before the operation to rule out an acute UTI. The injection itself was always carried out using a rigid cystoscope (17 or 21 French) in a specified manner [28]: the onabotulinumtoxin A was dissolved in 10 mL of sodium chloride. The application was carried out either under local anesthesia with 50 mL of lidocaine and a 20 min exposure time or under general anesthesia (by an anesthetist), according to the patient’s preferences. Twenty injections were made on the side walls of the bladder, the posterior wall, the base of the bladder, and in the trigonum vesicae, sparing the ostia areas. The intervention was always performed under perioperative antibiotic prophylaxis.

The automated Diary Pod was used again from the first postoperative day, ideally continued for 21 days. A control visit was made around 1–2 weeks after the injection day to rule out a UTI or significant residual urine formation.

Patients who showed a UTI in the control visit 1–2 weeks after the injection day were withdrawn from the study because this has a very significant and negative impact on the frequency of micturition. Likewise, patients who had not documented at least 2 preoperative and 7 postoperative days via the app could not be evaluated. Patients who had several gap days in a row in the visualized diary on the Minze Clinician Portal documentation were also excluded.

The ethics committee of the Charité Universitätsmedizin Berlin was consulted for the study.

Statistics: The mean voiding frequency value was calculated from the 3 days before the injection therapy to obtain a picture of the pre-therapeutic state that was as representative as possible. This mean represented the reference value for the post-therapeutic voiding frequencies. The values of the patients were compared with the baseline value (=average of the first 3 days) per single day (after injection) using a one-sample *t*-test. All statistical analyzes were performed using the program R-4.2.2. for Windows. Because of the several potential disruptive factors in interpreting the onset of action (e.g., UTI, missing documentation) and because of the relatively high effort for the patients (the motivation for app documentation was expected to decrease once the therapy was completed and the desired relief occurred), in the calculation of the patients to be included, we considered a high dropout rate. This study was not supposed to prove the effect of OnabotulinumtoxinA but to document the onset of the effect, and the number of completely evaluable patients was calculated accordingly.

## Figures and Tables

**Table 1:** Decrease in voiding frequency after injection therapy. The reduction in micturition in 24 h reached a trend on the 4th day and became significant on the 5th day. The reduction in voiding, separately during the day and at night, reached a significant level from the 7th day (not separately shown).

	Day	Frequency in 24 h (Mean ± SD)		*p*	Frequency Daytime (Mean ± SD)		*p*	Frequency Nighttime (Mean ± SD)		*p*
Average micturition BEFORE injection	−3 to −1	12.83	5.54		10.87	5.16		1.96	0.98	
Average micturition AFTER injection	1	12.29	4.39	0.767	10.14	4.02	0.636	2.14	0.90	0.689
	2	11.14	4.85	0.400	9.29	4.35	0.364	1.86	1.07	0.736
	3	9.56	4.45	0.060	7.89	4.31	0.070	1.67	0.87	0.282
	4	9.38	4.24	0.056	8.13	4.42	0.119	1.25	1.28	0.142
	5	9.17	3.19	0.038	7.67	4.03	0.107	1.50	1.22	0.363
	10	8.75	3.69	0.017	7.63	3.54	0.035	1.13	0.64	0.006
	15	7.17	2.04	0.001	6.00	2.10	0.002	1.17	0.75	0.042
	20	5.75	0.50	<0.001	4.25	0.96	0.001	1.50	1.29	0.049

## Data Availability

Not appliable.
